# Increased mRNA Stability and Expression Level of *Croceibacter atlanticus* Lipase Gene Developed through Molecular Evolution Process

**DOI:** 10.4014/jmb.2103.03011

**Published:** 2021-05-19

**Authors:** Han Byeol Jeong, Hyung Kwoun Kim

**Affiliations:** Division of Biotechnology, The Catholic University of Korea, Bucheon 14662, Republic of Korea

**Keywords:** Lipase, *Croceibacter atlanticus*, error-prone PCR, enzyme production

## Abstract

In order to use an enzyme industrially, it is necessary to increase the activity of the enzyme and optimize the reaction characteristics through molecular evolution techniques. We used the error-prone PCR method to improve the reaction characteristics of LipCA lipase discovered in Antarctic *Croceibacter atlanticus*. Recombinant *Escherichia coli* colonies showing large halo zones were selected in tributyrin-containing medium. The lipase activity of one mutant strain (M3-1) was significantly increased, compared to the wild-type (WT) strain. M3-1 strain produced about three times more lipase enzyme than did WT strain. After confirming the nucleotide sequence of the M3-1 gene to be different from that of the WT gene by four bases (73, 381, 756, and 822), the secondary structures of WT and M3-1 mRNA were predicted and compared by RNAfold web program. Compared to the mean free energy (MFE) of WT mRNA, that of M3-1 mRNA was lowered by 4.4 kcal/mol, and the MFE value was significantly lowered by mutations of bases 73 and 756. Site-directed mutagenesis was performed to find out which of the four base mutations actually affected the enzyme expression level. Among them, one mutant enzyme production decreased as WT enzyme production when the base 73 was changed (T→C). These results show that one base change at position 73 can significantly affect protein expression level, and demonstrate that changing the mRNA sequence can increase the stability of mRNA, and can increase the production of foreign protein in *E. coli*.

## 1. Introduction

Lipase is an industrially useful enzyme, and various enzymes have long been discovered from animals, plants, and microorganisms [1-3]. Recently, new lipases have been found in extreme regions of the earth [4-9]. In particular, enzymes that are active at low temperatures and resistant to organic solvents have been found from microorganisms isolated from Antarctica [4-7]. Psychrophilic enzymes generally have organic solvent stability, because they have high activity and stability, even under low water activity conditions [10-14]. This property is useful for utilizing lipase as a catalyst for various bioconversion reactions performed in organic solvents.

In order to use the enzymes found in nature for industrial use, they must be improved using molecular evolution methods. This is because they can only be used industrially if they have the temperature, pH, kinetic parameter, and stability suitable for diverse reaction conditions. Various molecular evolution techniques have been developed and used [15-20]. The molecular evolution of enzymes is performed by using random mutagenesis methods, such as the error-prone PCR and DNA shuffling, the rational design method based on the structure of proteins, and the site-directed random mutagenesis method, which mixes the above two methods.

On the other hand, research has been conducted to produce a large amount of recombinant enzymes in active form. The codon usage of *Escherichia coli* strain, which is the most widely used host, contains several rare codons. If the target lipase gene contains the rare codon of *E. coli*, this lipase cannot be well expressed in *E. coli* cells. In this case, the problem is solved by using the reinforced *E. coli* strain with a high level of the tRNA of the rare codon as host [21-25]. In addition, when the stability of mRNA in *E. coli* cells is low, the amount of protein synthesis is reduced, because the half-life of the mRNA is shortened. In this case, it is possible to increase the stability of the mRNA by changing the mRNA gene sequence, without changing the amino acid sequence of the protein [26]. Conversely, if too much proteins are produced in short time within *E. coli* cell cytoplasm, insoluble inclusion bodies are formed. To solve this problem, we can lower the culture temperature below 20°C, or reduce the IPTG concentration [27, 28]. Alternatively, when ethanol is added, proteins are often produced in soluble functional forms [29].

In this study, we used the error-prone PCR method to induce a random mutation of *Croceibacter atlanticus* lipase isolated from Antarctica, and produced mutant lipase with increased activity and improved expression level. The increase in the expression of mutant enzyme was explained by mRNA secondary structure prediction, minimum free energy calculation by the RNAfold web program. Four back mutations were performed in which the four base substitutions included in the M3-1 gene were changed to the original base by the site-directed mutagenesis method, respectively. In addition, the temperature and pH characteristics of the mutant enzyme were investigated.

## Materials and Methods

### Materials

Acetonitrile, ampicillin, tributyrin (TBN), p-nitrophenyl caprylate (pNPC), sodium dodecyl sulfate (SDS), 2-mercaptoethanol, Tris base, and bovine serum albumin (BSA) were purchased from Sigma-Aldrich Co. (USA). Ethanol was purchased from Merck Chemical Co. (Germany). Isopropyl β-D-thiogalactopyranoside (IPTG) was purchased from Duchefa Biochemie B.V. Co. (the Netherlands). X-gal was purchased from Promega (USA). LB broth was purchased from Difco Com. Taq polymerase and dNTP were purchased from Bioneer Co. (Korea). Oligonucleotides were purchased from Bionics (Korea).

### Production of LipCA Lipase

We received many strains isolated from Antarctic Ross Sea by the Korea Polar Research Institute. Among them, *Croceibacter atlanticus* (Stock No. 40-F12, 75°44'S, 176°57'E, depth 320 m), which formed the largest clear zone on TBN/Marine agar medium, was selected as the target strain. The lipase enzyme gene (*lipCA*) was found by shotgun cloning method as described previously [9].

The *lipCA* gene in pET22 vector was transformed into *E. coli* BL21 (DE3) cell. The transformed *E. coli* cells were inoculated into LB broth containing ampicillin (100 μg/ml) and incubated at 37°C until OD (600 nm) reached 0.5. IPTG (0.1 mM) was added and further incubated at 20°C for 20 h. After incubation, the bacteria were collected by centrifugation (3,500 ×*g*, 10 min) and resuspended with distilled water. The bacteria were disrupted by sonication, and then centrifuged (14,000 ×*g*, 10 min) to obtain a cell-free extract [9].

### Random Mutagenesis of LipCA Lipase

Random mutagenesis was used to improve the reaction characteristics and expression level of LipCA lipase. Specifically, error-prone PCR (epPCR) method was performed to induce the random mutagenesis of LipCA lipase. Compared to typical PCR reactions, the epPCR reaction was performed by reducing dATP by 1/10, and increasing Mg^2+^ ion concentration. That is, concentrations of 0.25 mM dTTP, dGTP, and dCTP, 0.025 mM dATP, and 3 mM MgCl_2_ were used. In order to clone the entire *lipCA* gene by ep-PCR, primers were prepared based on the nucleotide sequences at the beginning and end of the gene: forward primer 5'-GAT CAT ATG AAT CCT GCT GTT TTT-3', and reverse primer 5'-GAT AAG CTT TTA CAA CCG CTG AGC-3'. The other components were added to the reaction solution according to the general PCR method. The epPCR condition was as follows: initial denaturation at 95°C for 5 min, 25 cycles of denaturation at 95°C for 1 min, annealing at 40°C for 1 min, and polymerization at 73°C for 1 min, and final extension at 73°C for 7 min. After PCR reaction, agarose gel electrophoresis was performed, and PCR product was extracted from the gel. PCR product was digested with NdeI and HindIII restriction enzymes, ligated to pET22 vector, and transformed into *E. coli* DH5α. All recombinant plasmids were isolated at once from all *E. coli* colonies formed in agar medium, and transformed into *E. coli* BL21 (DE3). *E. coli* cells were incubated in TBN-LB agar medium for 2 days, and the mutant colonies with larger halos than WT were selected.

### Expression of Mutant Lipase Enzymes

Recombinant plasmids of WT and mutants were transformed into *E. coli* BL21 (DE3). *E. coli* cells were cultured in LB medium containing ampicillin (100 μg/ml) at 37°C. When the OD_600nm_ of the culture reached 0.5, 1 mM IPTG was added, and cultured at 20°C for 20 h. The culture was centrifuged (4,000 ×*g*, 10 min) to collect the *E. coli* cells, and suspended in 50 mM potassium phosphate buffer (pH 7.0). The suspended *E. coli* cells were disrupted by sonication, and centrifuged (12,000 ×*g*, 10 min), to obtain a cell-free extract (CFE). Protein concentration of CFE was measured by Bradford analysis at 595 nm, and protein profile of CFE was analyzed by SDS-PAGE.

### Lipase Activity Assay

Lipase activity assay was performed with pNPC as substrate. p-Nitrophenol (pNP) production amount was calculated by measuring the absorbance at 405 nm. One unit of lipase activity was defined as 1 μmol of pNP liberated per min under standard assay conditions (pH 8.0, 37°C, and 3 min). The assay was carried out in 1 ml reaction mixture containing 950 μl of 50 mM Tris-HCl (pH 8.0), 40 μl of ethanol, and 10 μl of 10 mM pNPC dissolved in acetonitrile. For enzyme dilution, 50 mM potassium phosphate buffer (pH 7.0) containing 1 mg/ml BSA was used.

### Characterization of the Mutant Lipase

In order to determine the optimum temperature of WT and M3-1 enzymes, their activities were measured using pNPC at 10°C intervals from (10 to 70) °. In addition, in order to determine the temperature stability, their residual activities were measured in the standard condition every 20 min, while incubating for 60 min at the temperature range (10-70) °.

In order to determine the optimum pH of WT and M3-1 enzymes, their activities were measured using pNPC at the pH range (6.5-10.5). In addition, in order to know the pH stability, the residual activity was measured in the standard condition, after incubating them for 60 min at the pH range (5.0-10.5).

### RNA Fold Prediction

The mRNA secondary structures of WT, M3-1, and back mutants (M3-1-73, M3-1-381, M3-1-755, and M3-1-822) were predicted using RNAfold web program (rna.tbi.univie.ac.at/cgi-bin/RNAWebSuite/RNAfold.cgi). This RNAfold web server predicts the secondary structures of single-stranded RNA or DNA sequences. The minimal free energy of optimal secondary structure and the thermodynamic ensemble structure were calculated for each mRNA. The thermodynamic parameter for bulge loop, free energy parameter for hairpin loop formation, internal loop free energy parameter, and free energy increment for multibranch loop initiation were used as presented in the previous literature [30-33].

### Back Mutation of M3-1 Lipase Gene

The QuikChange Lightning Site-Directed Mutagenesis kit (Agilent Technologies, USA) was used to induce four back mutations of the M3-1 lipase gene. In order to induce site-specific mutations, forward and reverse primers were prepared from the sequences around the target bases (Table 1), and PCR reaction was carried out according to the manufacturer’s instructions: initial denaturation at 95°C for 2 min, 18 cycles of denaturation at 95°C for 20 s, annealing at 60°C for 10 s, and polymerization at 68°C for 3 min 30 s, and final extension at 68°C for 5 min. The template DNA was removed with DpnI restriction enzyme at 37°C for 5 min. The resulting PCR product was transformed into *E. coli* XL10-Gold cells by heat shock reaction, incubated at 37°C for 1 h, and spread on LB agar medium. Four different plasmids (M3-1-73, M3-1-381, M3-1-756, and M3-1-822) with the desired back mutations were obtained from four different *E. coli* cells. These plasmids were transformed into *E. coli* BL21 (DE3), and lipase activity and expression level were investigated in each *E. coli* CFE.

## Results and Discussion

### Error-Prone PCR of *lipCA* Gene

**Fig. 1:**
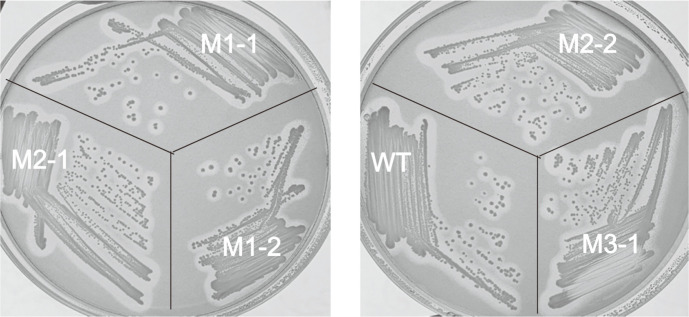
*E. coli* colonies carrying WT and mutant LipCA genes. *E. coli* BL21 (DE3) cells carrying WT and mutant LipCA genes were cultured in TBN-LB agar medium.

Random mutagenesis of the *lipCA* gene was performed for the purpose of improving the reaction characteristics and expression level of the LipCA lipase. To find the poor reaction condition that was far from optimal conditions, PCR reactions were performed under various conditions. Among them, it was decided to perform the epPCR reaction by reducing the amount of dATP to 1/10, compared to the other dNTPs, and increasing the MgCl_2_ concentration to 3.0 mM. After performing the reaction, by inserting the PCR product into the pET22 vector and transforming it into *E. coli* DH5α, we found more than 500 colonies. Recombinant plasmid library pool was isolated from all colonies at once, transformed into *E. coli* BL21 (DE3), spread on TBN-LB agar medium, and cultured at 37°C. Among the total colonies, the proportion of colonies forming halo in TBN-LB medium was confirmed to be about 10%. Five colonies forming the largest halo were selected among them, and named M1-1, M1-2, M2-1, M2-2, and M3-1 (Fig. 1), and the corresponding plasmids were purified.

### Expression of Mutant LipCA Lipase

*E. coli* BL21 (DE3) cells were transformed with WT and mutant genes, and IPTG induction experiments were performed, respectively. Then, protein concentration and lipase activity were measured in each *E. coli* CFEs (Table 2). WT showed a specific activity of 94.3 U/mg, while M1-1 showed a level of 90.6 U/mg, similar to that of WT. M1-2, M2-1, and M2-2 showed lower levels of (52.9, 55.2, and 39.1) U/mg, respectively, than WT activity. In the case of M3-1, the value was 292.7 U/mg, which was three times higher than the activity of WT.

**Fig. 2:**
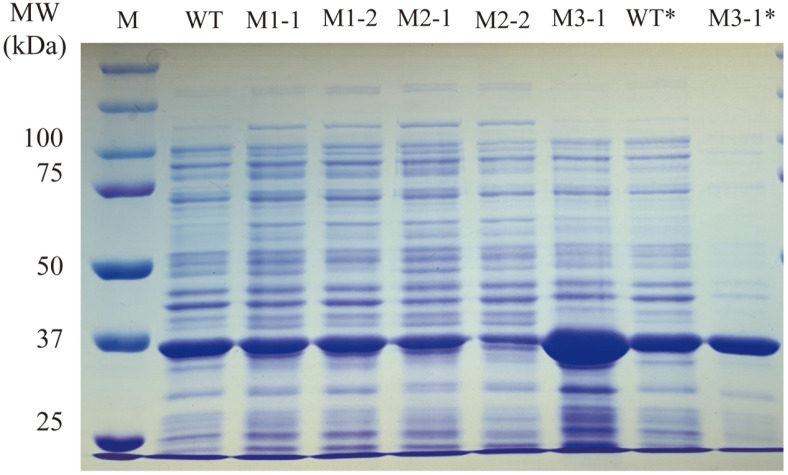
SDS-PAGE of cell-free extracts of *E. coli* cells carrying WT and mutant genes. The cell-free extracts of *E. coli* cells containing WT and mutant genes were analyzed by SDS-PAGE. M, protein size marker; WT* and M3-1*, CFE of 0.2 lipase unit.

As a result of SDS-PAGE, proteins of 35 kDa size were observed from all *E. coli* CFEs harboring WT gene and mutant genes. It was found that the amounts of target protein in M1-1, M1-2, and M2-1 were similar to that of WT, and lower in M2-2. However, in the case of M3-1, the amount of target protein was much higher than that of WT (Fig. 2). When WT and M3-1 CFE were used in the same lipase unit (0.2 U), it was confirmed that the thicknesses of the two target protein bands were similar on SDS-PAGE (Fig. 2). Through this, it was found that the specific activities of the two enzymes were similar.

We analyzed the nucleotide sequence of M3-1 gene. The M3-1 gene contained four base substitutions (T^73^ → C, A^381^ → G, T^756^ → C, and T^822^ → C). Among them, 3 substitutions were silent mutations, while the rest was missense mutation. Silent mutation means that the nucleotide sequence of the codon has changed, but the amino acid sequence is not changed due to codon degeneracy, and the missense mutation means that the amino acid sequence is changed by the change of the nucleotide sequence. Accordingly, one amino acid (Phe^25^→Leu) had been changed at the protein level. Since the lipase activity of M3-1 CFE was increased 3 times compared to that of WT CFE, and the production of target enzyme protein increased significantly, it was decided to investigate the enzyme properties and production of M3-1 lipase.

### Effect of Temperature on WT and M3-1 Lipases

**Fig. 3:**
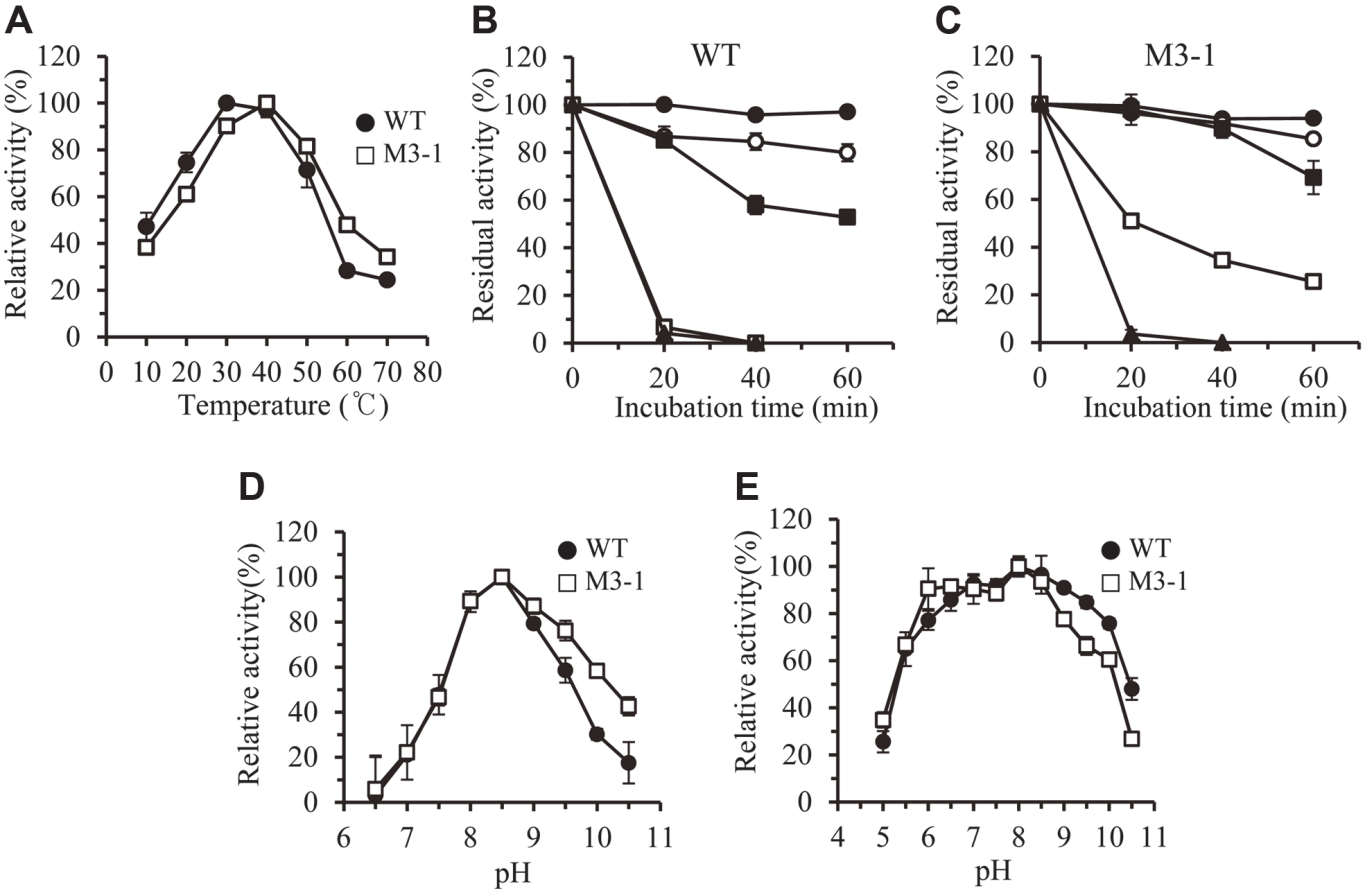
Effects of temperature and pH on LipCA lipase WT and M3-1. (**A**) The lipase activities of LipCA lipase WT and M3-1 were measured in the temperature range (10–70) °C. (**B**) and (**C**) The residual activities of WT and M3-1 were measured at intervals of 20 min under heat treatment for 60 min from (20 to 50) °C. ●, 20°C; ○, 30°C; ■, 40°C; □, 45°C; ▲, 50°C. (**D**) The lipase activities of WT and M3-1 were measured in the pH range (6.5–10.5). (**E**) The residual activities of WT and M3-1 were measured after pH treatment for 60 min from pH (5 to 10.5).

In order to investigate the effect of temperature on WT and M3-1 enzymes, lipase activity was measured at the temperature range (10 to 70) °. LipCA WT had an optimum reaction temperature of 30°C, and 50% activity was observed at 10°C. M3-1 enzyme had an optimum reaction temperature of 40°C, and its activity at higher temperatures was slightly increased, compared to WT (Fig. 3A). In terms of temperature stability, WT was stable at 20°C, and showed (80 and 50) % residual activity after 60 min at (30 and 40) °, respectively. Above 45°C, the activity completely disappeared after 20 min (Fig. 3B). In the case of M3-1 lipase, improved thermal stability was observed. After 60 min at (30 and 40) °, the residual activity was (90 and 70) %, respectively. Even at 45°C, a residual activity of 30% was observed after 60 min (Fig. 3C). Although this result cannot be clearly explained in terms of protein structure, it showed that the thermal stability of the enzyme can be changed by only one change in the amino acid sequence (Phe^25^ → Leu).

The optimum pH for both WT and M3-1 enzymes was found to be 8.5. When the pH was lower than 8.5, the activities of the two enzymes were similarly decreased (Fig. 3D). However, when the pH was higher than 8.5, the activity of M3-1 enzyme was slightly higher than that of WT enzyme. In the case of pH stability, the two enzymes had almost similar stability, *i.e.*, when treated for 1 h in the pH range (6-10), it was found that more than 60 % of residual activity was maintained (Fig. 3E).

### Comparison of WT and M3-1 Genes

As mentioned earlier, the specific activity of CFE of *E. coli* M3-1 was approximately 3 times higher than that of *E. coli* WT; as a result of SDS-PAGE analysis, it was found that this is because the expression level of M3-1 enzyme was significantly increased, compared to WT (Fig. 2). The codon usage of M3-1 and WT was compared to explain the increase in enzyme expression (Table 3). There are four different codons between WT and M3-1 (T^73^TT→CTT, GAA^381^→GAG, TTT^756^→TTC, GGT^822^→GGC). However, since the 8 codons are not rare codons of the *E. coli* host cell, the difference in codon usage could not explain the increase in expression level.

Another factor affecting the level of enzyme expression is the secondary structure of the mRNA. The secondary structure of mRNA of WT and M3-1 was predicted and compared using RNAfold web program, as described in the Materials and Methods part. The minimal free energies of MFE secondary structure and thermodynamic ensemble structure of WT and M3-1 were calculated, respectively (Table 4). Although the MFEs were slightly different between the two structures, the MFEs of M3-1 mRNA were found to be consistently lower than those of WT mRNA. This means that the structure of M3-1 mRNA is more stable than that of WT mRNA.

**Fig. 4:**
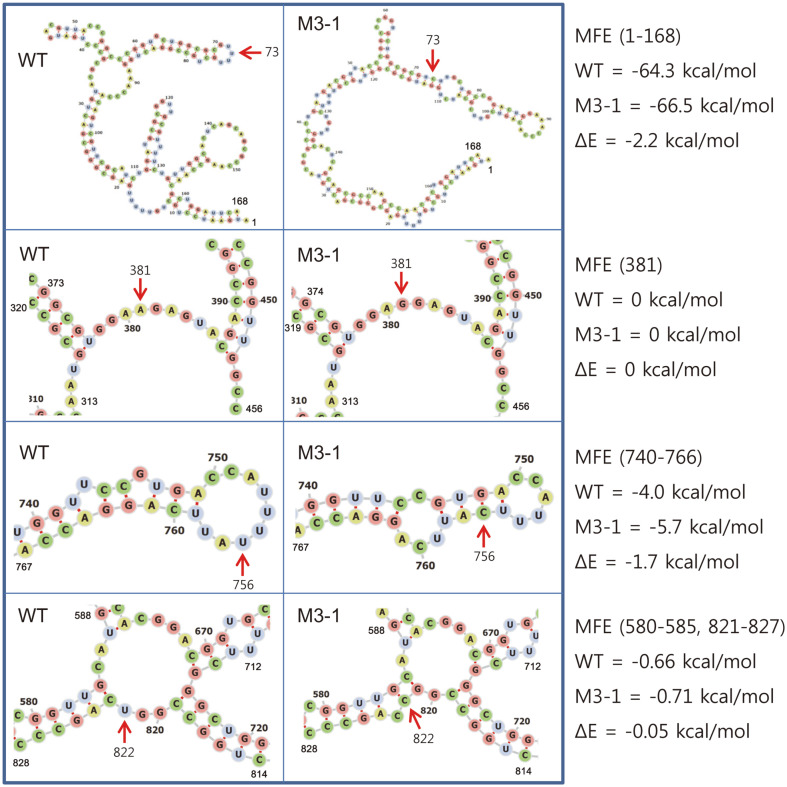
Prediction of the mRNA secondary structures of WT and M3-1. The sub-structures around the four base mutations (U^73^→C, A^381^→G, U^756^→C, and U^822^→C) in the predicted structure of WT and M3-1 mRNA were compared. Arrows indicate mutated base positions.

The structures around the four bases where mutations occurred were compared (Fig. 4). In the case of base 73, unlike the other three mutations, the mRNA structure was significantly changed by the mutation. In the case of WT, base 73 (U) was located in the hairpin loop, but in the case of M3-1, the base (C) formed a base pair to form a helix structure. Instead, two internal loops and a hairpin loop were additionally formed. According to this structural change, the MFE of M3-1 is 2.2 kcal/mol lower than that of WT in the base 1-168 section. In the case of base 381, both bases of WT and M3-1 were located in the multibranch loop. Therefore, it was calculated that there was no MFE difference between WT and M3-1 at this location. In the case of base 756, the base (U) of WT was located in the hairpin loop, but the base (C) of M3-1 was located in the helix structure. Instead, a smaller hairpin loop and an internal loop were formed. According to this structural change, the MFE of M3-1 is 1.7 kcal/mol lower than that of WT in the base 740-766 section. Finally, in the case of base 822, it can be seen that the base (U) of WT is located in the multibranch loop, but the base (C) of M3-1 is located in the helix with one bulge. According to this structural change, the MFE of M3-1 is 0.05 kcal/mol lower than that of WT in the base 580-585 and 821-827 section. In summary, the mutations of bases 73 and 756 significantly lowered the free energy of the mRNA, while the mutations of bases 381 and 822 maintained or slightly lowered the free energy, respectively.

To find out which of the four base mutations had the greatest influence on the MFE change, the four bases of M3-1 mRNA were changed to the same as WT, and the RNAfold web program was used to calculate the MFE of the back-mutated mRNA (Table 4). In the case of the mRNA that caused the two back mutations (M3-1-381 and M3-1-822), the MFEs were not significantly different from the MFE of M3-1. However, in the cases of M3-1-73 and M3-1-756, the MFEs were noticeably increased. This showed that the stabilities of these two mRNAs were reduced, compared to M3-1 mRNA. Accordingly, among the four nucleotide mutations, nucleotide mutation 73 and 756 seemed to have greater effect on the half-life of the mRNA molecules.

### Back Mutation of M3-1 Gene by Site-Directed Mutagenesis

**Fig. 5:**
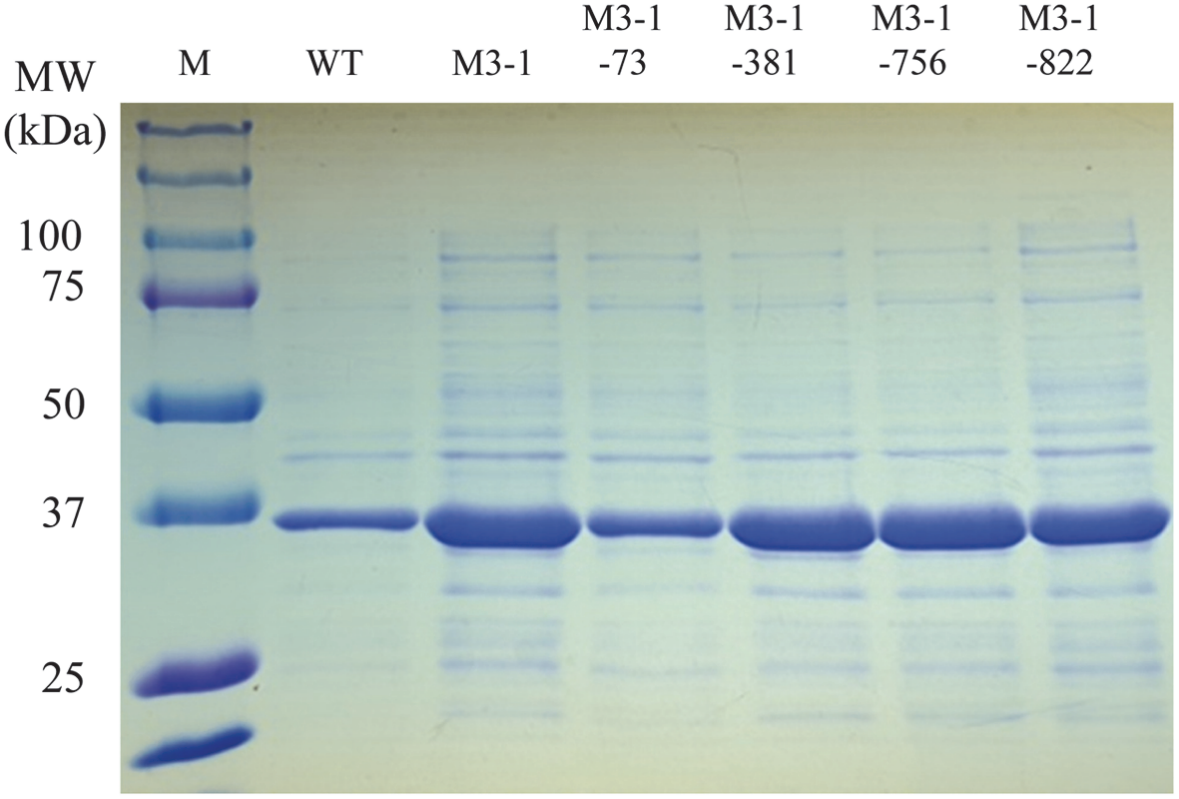
SDS-PAGE of the cell-free extracts of *E. coli* cells carrying WT, M3-1, and four back mutants. Cell-free extract of *E. coli* cells containing WT, M3-1, and four back mutant genes were analyzed by SDS-PAGE. M, protein size marker.

In order to determine which of the 4 bases of the M3-1 gene actually affected the RNA structure and expression level, each of the 4 bases of M3-1 gene was back-mutated in the same way as the WT bases. After expressing the back-mutated gene in *E. coli*, the expression level and specific activity of the four CFEs were compared with WT and M3-1 CFE (Fig. 5, Table 5). In the case of M3-1-73 CFE, which was expected to have a dramatic change in mRNA structure, the specific activity after back mutation was 128.8 U/mg, which was greatly reduced, making it similar to that of WT CFE. In the case of the other mutant enzymes (M3-1-381, M3-1-756, M3-1-822), (235.2, 276.7, and 225.2) U/mg activities were measured, respectively. These levels of activity were similar to the specific activity (292.7 U/mg) of M3-1 CFE. In addition, as a result of performing SDS-APGE, it was confirmed that the expression level of M3-1-73 was reduced, and that the level was similar to that of WT (Fig. 5). Based on these results, it can be confirmed that the change in the sequence of the 73 nucleotide of mRNA influenced the increase in the expression level of M3-1 gene.

The above experimental results suggest the following possibilities. In the case of genes that are not well expressed in *E. coli* cells, it is possible to predict the secondary structure of mRNA, and increase the amount of expression of the gene by changing the secondary structure, without changing the amino acid sequence. Accordingly, we provided the possibility of controlling the production of foreign proteins through mRNA engineering.

## Figures and Tables

**Table 1:** Primers used in back mutation.

Name	Primer (5’ – 3’)
F73	GCCCGGTGCTGGCGCGT*T*TTGCTGCCGGACTGGAAAC
R73	GTTTCCAGTCCGGCAGCAA*A*ACGCGCCAGCACCGGGC
F381	TACTGTGGGCGGCGTGGA*A*GAGTACGACCGGCTGTGC
R381	GCACAGCCGGTCGTACTC*T*TCCACGCCGCCCACAGTA
F756	CACTGGTTCCGTGACCATTT*T*ATTCAGGACCAGGCACTGAT
R756	ATCAGTGCCTGGTCCTGAAT*A*AAATGGTCACGGAACCAGTG
F822	CAACCCGGATCTGGCCGG*T*CAGCCCCCGGCAATTGTG
R822	CACAATTGCCGGGGGCTG*A*CCGGCCAGATCCGGGTTG

Primers were prepared to induce the 4 mutations from the M3-1 gene to the WT gene. Italics are bases for inducing back mutations, and underlined regions mean codons containing mutations.

**Table 2:** Lipase specific activity of CFEs of *E. coli* cell obtained by molecular evolution.

Mutant	Specific activity (U/mg)
WT	94.3
M 1-1	90.6
M 1-2	52.9
M 2-1	55.2
M 2-2	39.1
M 3-1	292.7

**Table 3:** Comparison of codons in WT and M3-1 genes.

Mutation position	WT			M3-1		

	Codon	Amino acid	Codon usage (%)^*^	Codon	Amino acid	Codon usage (%)^*^
73	TTT	Phe^25^	0.51	CTT	Leu^25^	0.10
381	GAA	Glu^127^	0.70	GAG	Glu^127^	0.30
756	TTT	Phe^252^	0.51	TTC	Phe^252^	0.49
822	GGT	Gly^274^	0.38	GGC	Gly^274^	0.40

*Codon usage in *E. coli* genes

**Table 4:** Minimal free energy of WT and mutant LipCA mRNA.

Gene	Minimal free energy of mRNA (kcal/mol)	

	MFE secondary structure	Thermodynamic ensemble structure
WT	-469.90	-485.72
M3-1	-474.30 (-4.4)**	-488.63 (-2.91)
M3-1-73*	-472.10 (-2.2)	-487.67 (-1.95)
M3-1-381*	-474.30 (-4.4)	-488.05 (-2.33)
M3-1-756*	-472.60 (-2.7)	-487.52 (-1.80)
M3-1-822*	-473.80 (-3.9)	-488.31 (-2.59)

Back mutation of M3-1 gene; M3-1-73 (C^73^→T), M3-1-381 (G^381^→A), M3-1-756 (C^756^→T), M3-1-822 (C^822^→T)

^†^The MFE structure of an RNA sequence is the secondary structure that contributes a minimum of free energy. This structure is predicted using a loop-based energy model and the dynamic programming algorithm introduced by Zuker *et al*. [33].

^‡^The thermodynamic ensemble is a statistical ensemble that is a probability distribution for the state of the system.

**Parentheses indicate the energy difference between mutant and WT.

**Table 5:** Lipase specific activity of CFEs of *E. coli* cells obtained by back mutation.

Mutant	Specific activity (U/mg)
WT	94.3
M3-1	292.7
M3-1-73	128.8
M3-1-381	235.2
M3-1-756	276.7
M3-1-822	225.2

## References

[1] Hitch TCA, Clavel T (2019). A proposed update for the classification and description of bacterial lipolytic enzymes. Peer J..

[2] Widmann M, Juhl PB, Pleiss J (2010). Structural classification by the lipase engineering database: a case study of Candida antarctica lipase A. BMC Genomics.

[3] Fischer M, Pleiss J (2003). The lipase engineering database: a navigation and analysis tool for protein families. Nucleic Acids Res..

[4] Hashim NHF, Mahadi NM, Illias RM, Feroz SR, Bakar FDA, Murad AMA (2018). Biochemical and structural characterization of a novel cold-active esterase-like protein from the psychrophilic yeast *Glaciozyma antarctica*. Extremophiles.

[5] Salwoom L, Salleh AB, Convey P, Mohamad Ali MS (2019). New recombinant cold-adapted and organic solvent tolerant lipase from psychrophilic *Pseudomonas* sp.LSK25, isolated from Signy Island Antarctica. Int. J. Mol. Sci..

[6] Park SH, Kim SJ, Park S, Kim HK (2019). Characterization of organic solvent-tolerant lipolytic enzyme from *Marinobacter lipolyticus* isolated from the Antarctic Ocean. Appl. Biochem. Biotechnol..

[7] Maharana AK, Singh SM (2018). A cold and organic solvent tolerant lipase produced by Antarctic strain *Rhodotorula* sp.Y-23. J. Basic Microbiol..

[8] Won SJ, Jeong HB, Kim HK (2020). Characterization of novel salt-tolerant esterase isolated from the marine bacterium *Alteromonas* sp.39-G1. J. Microbiol. Biotechnol..

[9] Park CG, Kim HK (2018). Production, immobilization, and characterization of *Croceibacter atlanticus* lipase isolated from the Antarctic Ross sea. Microbiol. Biotechnol. Lett..

[10] Hough DW, Danson MJ (1999). Extremozymes. Curr. Opin. Chem. Biol..

[11] Adams MW, Perler FB, Kelly RM (1995). Extremozymes: expanding the limits of biocatalysis. Biotechnology.

[12] Gomes JI, Steiner W (2004). The biocatalytic potential of extremophiles and extremozymes. Food Technol. Biotechnol..

[13] Trincone A (2011). Marine biocatalysts: enzymatic features and applications. Mar. Drugs.

[14] Demirjian DC, Morı's-Varas F, Cassidy CS (2001). Enzymes from extremophiles. Curr. Opin. Chem. Biol..

[15] Dror A, Shemesh E, Dayan N, Fishman A (2014). Protein engineering by random mutagenesis and structure-guided consensus of *Geobacillus stearothermophilus* lipase T6 for enhanced stability in methanol. Appl. Environ. Microbiol..

[16] Bilyk B, Weber S, Myronovskyi M, Bilyk O, Petzke L, Luzhetskyy A (2013). In vivo random mutagenesis of streptomycetes using mariner-based transposon Himar1. Appl. Microbiol. Biotechnol..

[17] Mabizela-Mokoena NB, Limani SW, Ncube I, Piater LA, Litthauer D, Nthangeni MB (2017). Genetic determinant of *Bacillus pumilus* lipase lethality and its application as positive selection cloning vector in *Escherichia coli*. Protein Expr. Purif..

[18] Guan L, Gao Y, Li J, Wang K, Zhang Z, Yan S (2020). Directed evolution of *Pseudomonas fluorescens* lipase variants with improved thermostability using error-prone PCR. Front. Bioeng. Biotechnol..

[19] Głód D (2017). Modification of fatty acid selectivity of *Candida antarctica* lipase A by error-prone PCR. Biotechnol. Lett..

[20] Panda AK, Bisht SPS, Panigrahi AK, De Mandal S, Kumar NS (2016). Cloning and in silico analysis of a high-temperature inducible lipase from *Brevibacillus*. Arab. J. Sci. Eng..

[21] Kane JF (1995). Effects of rare codon clusters on high-level expression of heterologous proteins in *Escherichia coli*. Curr. Opin. Biotechnol..

[22] Schwersensky M, Rooman M, Pucci F (2020). Large-scale in silico mutagenesis experiments reveal optimization of genetic code and codon usage for protein mutational robustness. BMC Biol..

[23] Zhou WJ, Yang JK, Mao L, Miao LH (2015). Codon optimization, promoter and expression system selection that achieved high-level production of *Yarrowia lipolytica* lipase in Pichia pastoris. Enzyme Microb. Technol..

[24] Ghahremanifard P, Rezaeinezhad N, Rigi G, Ramezani F, Ahmadian G (2018). Designing a novel signal sequence for efficient secretion of *Candida antarctica* lipase B in *E. coli*: the molecular dynamic simulation, codon optimization and statistical analysis approach. Int. J. Biol. Macromol..

[25] Chakraborty S, Nag D, Mazumder TH, Uddin A (2017). Codon usage pattern and prediction of gene expression level in *Bungarus* species. Gene.

[26] Mauger DM, Cabral BJ, Presnyak V, Su SV, Reid DW, Goodman B (2019). mRNA structure regulates protein expression through changes in functional half-life. Proc. Natl. Acad. Sci. USA.

[27] Larsen MW, Bornscheuer UT, Hult K (2008). Expression of *Candida antarctica* lipase B in *Pichia pastoris* and various *Escherichia coli* systems. Protein Expr. Purif..

[28] Gutiérrez-González M, Farías C, Tello S, Pérez-Etcheverry D, Romero A, Zúñiga R (2019). Optimization of culture conditions for the expression of three different insoluble proteins in *Escherichia coli*. Sci. Rep..

[29] Thomas JG, Baneyx F (1996). Protein misfolding and inclusion body formation in recombinant *Escherichia coli* cells overexpressing heat-shock proteins. J. Biol. Chem..

[30] Gruber AR, Lorenz R, Bernhart SH, Neuböck R, Hofacker IL (2008). The Vienna RNA Websuite. Nucleic Acids Res..

[31] Lorenz R, Bernhart SH, Höner zu Siederdissen C, Tafer H, Flamm C, Stadler PF (2011). "ViennaRNA Package 2. Algorithms for Molecular Biology..

[32] Mathews DH, Disney MD, Childs JL, Schroeder SJ, Zuker M, Turner DH (2004). Incorporating chemical modification constraints into a dynamic programming algorithm for prediction of RNA secondary structure. Proc. Natl. Acad. Sci. USA.

[33] Zuker M, Stiegler P (1981). Optimal computer folding of large RNA sequences using thermodynamics and auxiliary information. Nucleic Acid Res..

